# The *3Ds* in virus‐like particle based‐vaccines: “*Design, Delivery and Dynamics*”

**DOI:** 10.1111/imr.12863

**Published:** 2020-05-30

**Authors:** Mona O. Mohsen, Gilles Augusto, Martin F. Bachmann

**Affiliations:** ^1^ Interim Translational Research Institute “iTRI” National Center for Cancer Care & Research (NCCCR) Doha Qatar; ^2^ Department of BioMedical Research Immunology RIA University of Bern Bern Switzerland; ^3^ Jenner Institute Nuffield Department of Medicine University of Oxford Oxford UK

**Keywords:** delivery, design, dynamics, vaccine, virus‐like particles

## Abstract

Vaccines need to be rationally **designed** in order be **delivered** to the immune system for maximizing induction of **dynamic** immune responses. Virus‐like particles (VLPs) are ideal platforms for such 3D vaccines, as they allow the display of complex and native antigens in a highly repetitive form on their surface and can easily reach lymphoid organs in intact form for optimal activation of B and T cells. Adjusting size and zeta potential may allow investigators to further fine‐tune delivery to lymphoid organs. An additional way to alter vaccine transfer to lymph nodes and spleen may be the formulation with micron‐sized adjuvants that creates a local depot and results in a slow release of antigen and adjuvant. Ideally, the adjuvant in addition stimulates the innate immune system. The dynamics of the immune response may be further enhanced by inclusion of Toll‐like receptor ligands, which many VLPs naturally package. Hence, considering the 3Ds in vaccine development may allow for enhancement of their attributes to tackle complex diseases, not usually amenable to conventional vaccine strategies.

**Abbreviations**
a.a.: amino acids
Ab: antibody
APC: antigen‐presenting cell
BCG: bacillus Calmette‐Guérin
BCR: B‐cell receptor
CCMV: cowpea chlorotic mosaic virus
CP: coat protein
CPMV: cowpea mosaic virus
CTL: cytotoxic T‐lymphocyte
CuMV: cucumber mosaic virus
Cys: cysteine
DBCO: dibenzocyclooctyne
DC: dendritic cell
eRNA: eukaryotic RNA
FDC: follicular dendritic cell
FHV: flock house virus
GMP: good manufacturing practice
HBV: hepatitis B virus
HEV: hepatitis E virus
HIV: human immunodeficiency virus
HLA‐DR: human leukocyte antigen‐DR isotype
HPV: human papillomavirus
IL: interleukin
IM: intramuscular
LCMV: lymphocytic choriomeningitis virus
LN: lymph node
Lys: lysine
MAVS: mitochondrial antiviral signaling
MCT: microcrystalline tyrosine
MHC‐I: major histocompatibility class I
MHC‐II: major histocompatibility class II
mRNA: messenger RNA
NA: nucleic acid
NHS: N‐hydroxysuccinimide
PASP: pathogen‐associated structural pattern
pRNA: prokaryotic RNA
RIG‐1: retinoic acid–inducible gene‐like receptor
rRNA: ribosomal RNA
a: N‐succinimidyl S‐acetylthioacetate
SC: subcutaneous
SH: sulfhydryl
SMPH: succinimidyl 6‐((beta‐maleimidopropionamido)hexanoate)
T: triangulation
TLR: Toll‐like receptor
TMV: tobacco mosaic virus
tRNA: transfer RNA
TT: tetanus toxoid
VLP: virus‐like particle

## 1 BRIEF LOOK AT THE HISTORY OF VACCINES: WHERE WE ARE COMING FROM

Viruses are highly evolved and adapted supramolecular entities that take advantage of their host and can be strongly pathogenic.^1^ Viruses can cause pathogenesis by their entry and replication in host cells. Damage is usually caused by lytic activity of the virus or killing infected cells mediated by the immune cells, resulting in excessive cell death and inflammation. Hence, early stop of viral spread is the goal of most vaccinations.^2^

The practice of variolation, also called “inoculation or insertion,” was used in China in the 10th century to immunize people against smallpox. Live, virulent viruses were collected from scabs of infected victims and inserted into the skin of healthy individuals. The concept of variolation against smallpox spread further to India in the 17th century followed to southwest Asia and Balkans^3^, ^4^ and even to London by Lady Mary Montagu.^5^ Variolation was considered the sole practice to induce protection against smallpox until 1796 when Edward Jenner tested his hypothesis that pus from blisters from cowpox‐infected milkmaids can protect against smallpox. Jenner inoculated an 8‐year‐old boy, James Phipps, in both arms with this cow‐derived virus, causing the boy to develop some mild symptoms. Later, he challenged the boy with smallpox (by way of variolation), and there were no signs of disease.^6^, ^7^ The method was termed vaccination from the Latin word *vacca,* which means *cow*, and was much safer with milder reactions than variolation (which caused up to 1% death) and no risk of disease transmission.^8^ Mass vaccination against smallpox, finally pioneered by the World Health Organization, resulted in eventual global eradication of the disease by 1979. While the world has seen many more vaccines with improved safety profiles, no subsequent vaccine ever reached the efficacy of vaccinia‐based vaccination. The next vaccine hallmark occurred with the revolutionizing work of Louis Pasteur for the development of an attenuated cholera vaccine in 1897 and inactivated anthrax vaccine in 1904 for humans. Both were produced in laboratories rather than by straight isolation from humans or cows as it was the case for vaccinia virus. Vaccination against viruses represented more of a challenge to Pasteur than vaccination against bacteria, most likely because viruses required cell culture techniques not yet developed. Nevertheless, Pasteur could successfully protect dogs from rabies as well as a 9‐year‐old boy who had been severely attacked by infected dogs with a vaccine that was, however, produced in the brain of infected rabbits.^9^ In the late 19th century, a vaccine against plague (*Yersinia pestis*) was started by Alexandre Yersin based on killed whole‐cell bacteria. However, the vaccine proved less efficacious against the most severe pneumonia form of plague.^10^ For this reason and due to re‐emergence of plague, efforts are currently made to develop more effective vaccines against plague based on attenuated bacteria and recombinant proteins.^11^ The development of a vaccine against tuberculosis occurred during the period of 1890 to 1950. At the end of the 19th century, Calmette and Guérin started their research for a vaccine against tuberculosis at the Pasteur Institute, resulting in the Bacillus Calmette‐Guérin (BCG) vaccine strain.^12^ The efficacy of this BCG vaccine is still controversial, despite the fact that live BCG is in use as a vaccine against TB in humans in some parts of the world where it seems to attenuate severe forms of TB.^13^, ^14^

The Salk vaccine “inactivated poliovirus” was developed by Jonas Salk in the 1950s, while the Sabin vaccine “live attenuated poliovirus” was advanced by Albert Sabin for oral administration. Though highly efficacious, the Sabin vaccine is only used rarely nowadays due to the potential to revert to a virulent form or cause disease in immunocompromised individuals. Mass immunization against poliovirus resulted in the eradication of the disease in most parts of the world.^15^ The widely used “MMR” vaccines against “measles, mumps, and rubella” have been developed using attenuated viruses.^16^ The MMR vaccine has massively reduced the presence of these viruses in our society and, in terms of safety and, portrays a positive benefit‐risk profile.^17^ Vaccines may not only be used to protect against different pathogens, but they also play an important role in the treatment of allergies.^18^ Furthermore, and perhaps with the brightest future, a large number of therapeutic vaccines are currently developed for the treatment of cancer and other non‐communicable diseases, such as hypertension, psoriasis, asthma, and type II diabetes.

Taken together, traditional vaccines (inactivated or attenuated pathogens or toxins) have shown high immunogenicity and ability to stimulate the innate and adaptive immune system conferring long‐term memory and protection. Such high immunogenicity, particularly where viral vaccines are concerned, is due to several factors such as the ability of the pathogen to replicate, the repetitive surface geometry of the virus and its particulate nature.^19^ Yet, several drawbacks in the traditional vaccines have been raised by policymakers, including, but not limited to, safety issues from using the whole pathogen and the difficulty in mass production.

The 21st century has witnessed an advancement in molecular genetics, microbiology, immunology, and genomics techniques in applied vaccinology. Scientists have taken advantage of viruses by making them invaluable sources for generating safe and novel nanoscale scaffolds termed virus‐like particles (VLPs). VLPs are multiprotein structures that resemble viruses in many characteristics.^20^ The most important difference is that VLPs lack any genetic material and therefore cannot replicate in host cells.^21^ Accordingly, VLPs serve as a premium vaccine platform overcoming several limitations of traditional vaccines such as poor definition, safety, or manufacturing obstacles. VLP platforms have led to considerable success in developing vaccines against human papillomavirus (HPV), hepatitis B virus (HBV), hepatitis E virus (HEV) as well as against malaria (for a review, see Ref. 21). Several other VLP‐based vaccines are in preclinical evaluation or undergoing clinical trials. In this review, we are discussing the basic 3Ds employed for VLP‐based vaccine development: ***design*, *delivery*, and *dynamics*.**

## 2 DESIGN

### 2.1 VLP geometry

Crick and Watson have stated in 1956 that “it is a striking fact that almost all small viruses are rods or spheres.” “These shells are constructed from a large number of identical protein molecules, of small or moderate size, packed together in a regular manner”.^22^ A key reason for such an arrangement is the small genomes of many viruses, in particular RNA viruses, which have no space to encode multiple proteins to build up a complex surface. As repetitiveness is unavoidable, the quasicrystalline nature of VLPs serves as a potent pathogen‐associated structural pattern (PASP) recognized by both innate and adaptive immune systems.^23^

The majority of viruses have a coat or a nucleocapsid consisting of multiple copies of single or just a few different kinds of structural proteins arranged in icosahedral or helical shapes. Coat proteins (CPs) can self‐assemble in artificial expression systems or in vitro spontaneously with or without the aid of nucleic acids (NA) or scaffold proteins. According to purely mathematical principles, an icosahedron is built from 60 identical units arranged in 5‐3‐2 symmetry which is used to describe the rotation possibilities of an icosahedron around an axis.^24^, ^25^

**FIGURE 1:**
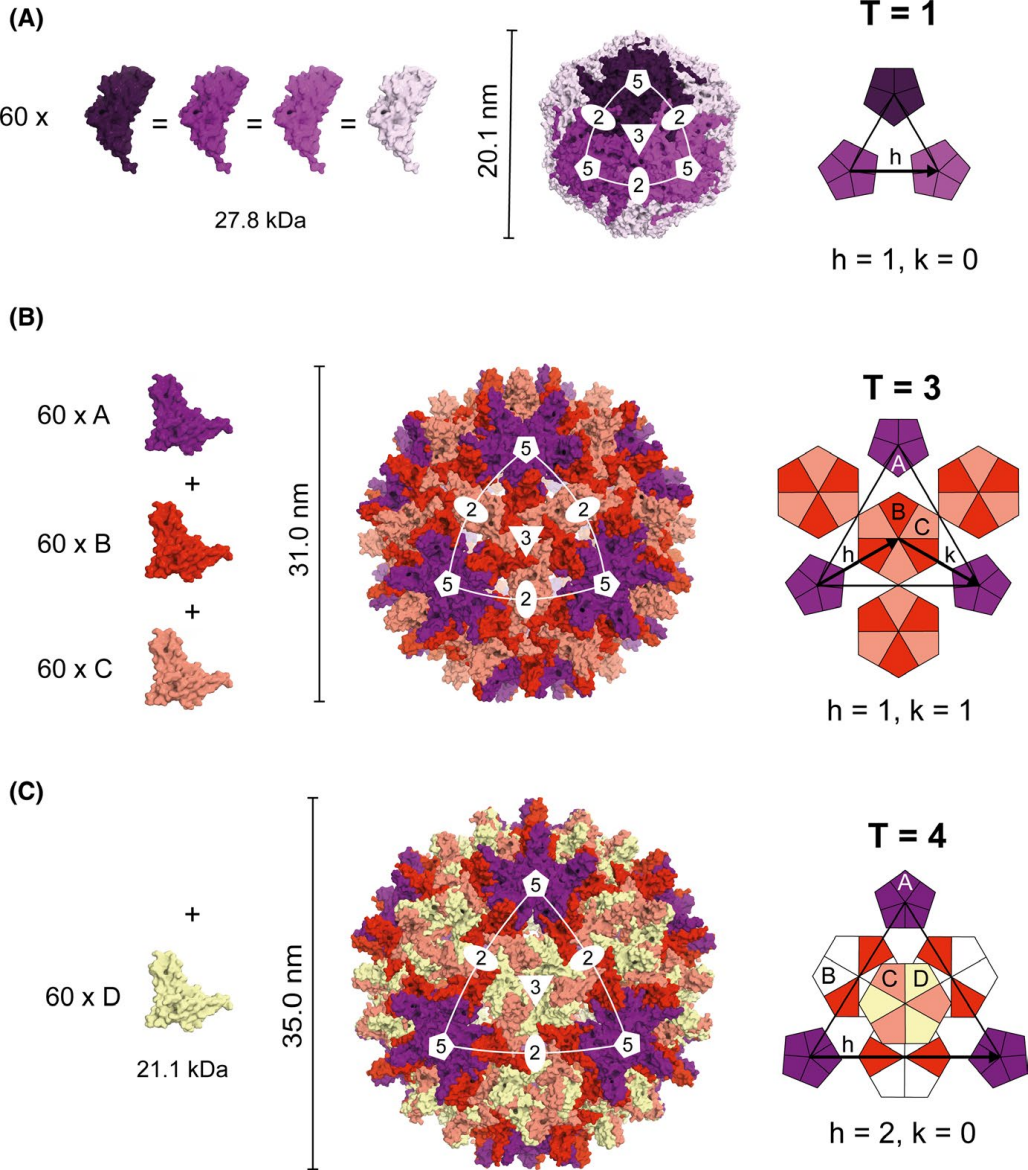
Geometry of icosahedral viral particles. Three typical geometries of icosahedral viral capsids. From left to right, the panels show the color codes for the subunits and their approximate molecular weight; the external view of the viral capsid with the symmetry axes drawn over the icosahedron facet; and the disposition of the subunits over the hexagonal plan, with the triangulation path (h and k indexes) illustrated by black arrows. Vertical bars illustrate the approximate diameter of the particle. A, Capsid of porcine circovirus serotype 2 (PCV2) observed in *T* = 1 configuration. PCV2 particles are formed by 60 identical subunits combined into 12 pentamers (illustrated here in different shades of purple just for clarity). From PDB 3JCI. B, Hepatitis B virus's (HBV's) capsid observed in T3 configuration. When in this configuration, HBV particles are formed by 60 of each subunit A, B, and C. From PDB 6BVN. c, HBV can also be observed in a T4 configuration, when it has an extra subunit D incorporated into its capsid arrangement. From PDB 6BVF

Indeed, Crick and Watson predicted that icosahedral viruses must have at least 60 subunits.^22^ It has also been shown that an icosahedron with certain multiples of 60 subunits can be formed by incorporating subunits arranged in pentamers and hexamers.^26^ However, in this case all subunits cannot be structurally identical as different environments (3D structures) exist within pentamers and hexamers. In practice, often a single CP species can adopt minor structural changes required for pentameric or hexameric contacts, a concept called quasi‐equivalence. The number and arrangement of the different subunits of the virus coat can be classified using the quasi‐equivalence theory described by Casper and Klug in 1962 based on triangulation.^27^ The triangulation number (*T*) of the coat is determined by two integers *h* and *k* and is defined as *T* = *h* ^2^+*hk* + *k* ^2^.^27^ Essentially, *h* and *k* correspond to the number of steps, which must be traveled along two axes in a hexagonal grid across the centers of pentameric and hexameric subunits in order to reach one vertex of icosahedron from another. Figure 1 illustrates icosahedral VLPs with *T* = 1 coat symmetry using porcine circovirus serotype 2 (PCV2) (Figure 1A) and *T* = 3 or *T* = 4 coat symmetry using HBV core (HBc) (Figure 1B,C). Hepatitis B virus has an unusual feature that it can be found in both *T* = 3 or *T* = 4 forms,^28^ albeit the infectious virions appear to be *T* = 4 only.^29^, ^30^

It is interesting to note that most artificially designed synthetic VLPs consisting of a trimerizing and pentamerizing alpha‐helical domain form *T* = 1 particle with 60 spikes. Due to several reasons listed in Table 1, small synthetic *T* = 1 VLPs may often be not ideal for a vaccine platform and *T* = 3 particles may be more favorable.

**TABLE 1:** Comparison between *T* = 1 and *T* = 3 VLPs

Characteristic	*T* = 1 VLPs	*T* = 3 VLPs	Reference
B‐cell receptor (BCR) cross‐linking	Cross‐linking 60 BCRs on B cells is likely at the threshold for optimal immune response	Cross‐linking 180 BCRs on B cells results in a strong immune response	31
Distance between each displayed epitope on the surface of VLPs	The distance between each displayed epitope on the surface of *T* = 1 VLPs is usually less than 5‐10 nm	The distance between each displayed epitope on the surface of *T* = 3 VLPs is approximately 5‐10 nm, which is ideal for BCR cross‐linking and B cell activation	32
Interior cavity	Small cavity	Large cavity that allows packaging of large amounts of cargo such as nucleic acids, and Toll‐like receptor ligands for optimal activation of B and/or T cells	28, 33, 34

Abbreviation: B‐cell receptor; VLPs, virus‐like particles

Examples of VLPs with subunits arranged in *T* = 3 configuration are ssRNA bacteriophages such as MS2, AP205, and Qβ as well as several plant viruses such as cowpea chlorotic mottle virus (CCMV) and cucumber mosaic virus (CuMV). There is an interesting difference between the ssRNA bacteriophages and ssRNA plant viruses. First, the folds of the CPs are entirely different in both cases, CPs of plant viruses have classical jelly‐roll β‐barrel fold,^35^ while CPs of ssRNA phages have a completely unique fold, not observed in any other proteins. Secondly, the ssRNA‐phage VLPs have subunit proteins of approximately 14 kDa^36^, ^37^ while VLPs derived from plant viruses have subunit proteins of approximately 24 kDa.^35^ The likelihood to have a good T_H_ cell epitope in a given protein is proportional to the number of amino acids (a.a.) present in the protein. VLPs derived from plant viruses have a longer epitope sequence and therefore may be superior to bacteriophage VLPs in activating T_H_ cells even though both have *T* = 3 symmetry.

In general, icosahedron architecture is more prevalent than the helical shape. The definition of helical symmetry covers a broad area of geometries such as stacked rings, rods, or spring‐shaped coils.^38^ The helical symmetry of VLPs can be measured using the formula *P* = *μ* × *ρ* where *P* refers to the pitch of the helix, *μ* is the number of structural units per turn of the helix, and *ρ* is the axial rise per subunit. A well‐known example of helical rod‐shaped VLPs with hollow central channel is the tobacco mosaic virus–like particles.^39^ Interestingly, icosahedral VLP may easily be manipulated to form rod‐shaped structures by introducing few mutations. It has been shown previously that the icosahedral Qβ‐VLPs would assemble in rod‐like particles after mutating five a.a. residues in the FG loop of the CP.^40^ Presumably, this happens because of overproduction of hexamers over pentamers which leads to formation of prolate icosahedrons, or rods, rather than regular icosahedrons, as demonstrated also in the case when RNA phage coats of genogroups I and II are mixed together and reassembled in vitro.^41^ Tobacco mosaic virus–based VLPs have proven in some studies to be an effective platform for the display of epitopes, successfully eliciting immune response against different target pathogens.^42^, ^43^

Remarkably, the CPs of RNA phages can self‐assemble not only into the classical *T* = 3 particles and the above‐mentioned rod‐like prolate icosahedrons, but also into *T* = 1^44^ and *T* = 4 particles,^45^, ^46^ demonstrating the high plasticity of the assembly process. Generally, manipulating VLPs' structure allows to study the impact of size on drainage dynamics and magnitude of induced immune responses with one and the same VLP monomer, an avenue of research we are currently following.

### 2.2 Binding and decorating functional molecules or ligands to VLPs

**FIGURE 2:**
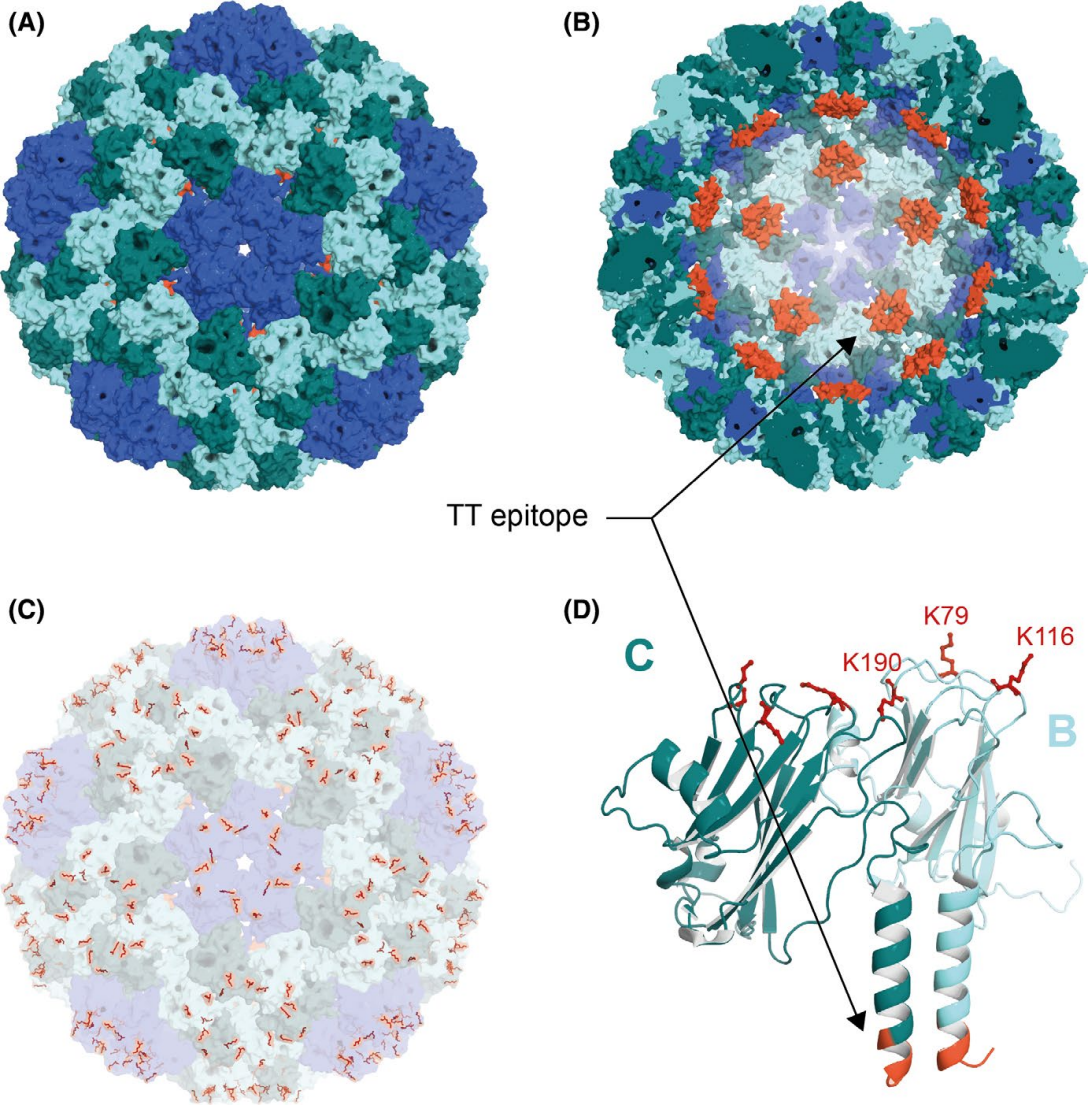
Cucumber mosaic virus (CuMV)–derived virus‐like particle fused to universal tetanus toxoid (TT) T‐cell epitope. General structural features of CuMV_TT_. A, Surface representation of viral capsid. Its *T* = 3 symmetry is formed by pentamers of subunit A (blue) and hexamers of subunits B (dark cyan) and C (light cyan). B, Cross section of the particle as shown in (A). The N‐termini of both subunits B and C are colored orange to indicate the points of insertion of the tetanus toxoid epitope. C, The same view as in (A) but with the surface lysine residues liable to conjugation highlighted in red. D, Cartoon representation of a dimer formed by subunits B and C. All images generated from PDB 1F15

Bioorganic chemistry offers different artificial ways to modify proteins and other molecules. For successful chemical transformation of the biological molecules in VLP‐based vaccine development, several aspects should be considered: (a) The reaction should be efficient in an aqueous solvent with physiological pH values; (b) low concentrations of non‐toxic reagents should be used; and (c) the tertiary and quaternary structure of the antigen and VLP should be preserved. Using different chemical modification strategies, we have reached numerous proofs of concept in humans and animals. For example, vaccination with Qβ‐VLPs displaying angiotensin II on the surface resulted in reduced blood pressure.^47^ A similar vaccine displaying nicotine has reduced smoking^48^ and a vaccine displaying full‐length interleukin (IL)‐1β reduced some signs of type II diabetes.^49^, ^50^ A vaccine against Alzheimer's disease displaying the first six a.a. of Aβ1‐42 reduces plaque load in mice and humans^51^, ^52^ and is now in final clinical trials. The platform has also been tested for classical prophylactic vaccines and display of the globular domain of influenza HA resulted in a vaccine candidate inducing protective titers in immunized humans.^53^, ^54^ More recently, we have moved to treat companion animals using a new, immunologically improved platform based on CuMV incorporating a universal T cell epitope derived from tetanus toxoid (TT) termed CuMV_TT_‐VLPs.^55^ The modified CuMV_TT_‐VLPs is a *T* = 3 VLP identical to the parent CuMV‐VLPs where subunits A are arranged in pentamers of five‐fold symmetry and three dimers of subunits B and C are arranged in hexamers in three‐fold symmetry^55^ as illustrated in Figure 2A and Video S1.

The newly developed platform is thought to enhance the interaction between T_H_ cells and B cells under normal as well as more challenging conditions such as those found in aged patients. This is due to the fact that preexisting immunity to the chosen TT epitope is very broad in humans (and animals) as the peptide binds to essentially all human leukocyte antigen‐DR isotype molecules and everybody has been immunized many times against TT.^56^, ^57^ The TT epitope has been incorporated by genetic fusion to the CuMV viral envelope protein such that the icosahedral particle maintains its capacity to self‐assemble without altering its *T* = 3 icosahedron geometry and without exposing the peptide on the VLP surface, avoiding interference of TT‐specific antibodies (Figure 2B). This has been achieved by replacing the first 12 N‐terminal a.a. with the TT epitope. The resulting CuMV_TT_‐VLP platform has several advantages, including the following: (a) It is adaptable to clinical applications; (b) CuMV_TT_‐VLP monomers have a size of 24 kDa, which can serve as an additional source of  T_H_ cell epitope; (c) the VLPs incorporate RNA derived from the *E coli* production strain serving as a Toll‐like receptor (TLR)7/8 agonist; (d) the VLPs are stable for long time periods at 4°C and even 20°C; and (e) the platform is safe and highly immunogenic in mice, rabbits, dogs, cats, and horses and therefore is also expected to offer a good safety profile and immunogenicity in humans.^55^, ^58^

Using this new CuMV_TT_ platform, we have generated a proof of concept for different preclinical vaccines as listed in Table 2. The most popular targets for modification on VLPs are accessible lysine residues (Lys) on their surface (Figure 2C). Lysine residues contain primary amines and can be modified at physiological pH values. N‐hydroxysuccinimide (NHS) esters are commercially available such as the commonly used cross‐linker SMPH [succinimidyl 6‐((beta‐maleimidopropionamido)hexanoate)]. The heterobifunctional SMPH cross‐linker has a reactive NHS group on one end and a maleimide group on the other end. The NHS group reacts with the Lys residue on VLPs while the maleimide group reacts with a free sulfhydryl (SH) group in the target molecule forming a stable amide and thioether bond, respectively. SH exists on the side chain of cysteine residues (Cys) and can be incorporated easily in the target molecule for effective cross‐linking.^38^ To link target proteins to VLPs without introducing free Cys residues, the SH group can be introduced in a protected form using the chemical cross‐linker SATA (N‐succinimidyl S‐acetylthioacetate) which then can be exposed after treatment with hydroxylamine for conjugation reactions. SATA also contains an NHS ester facilitating the formation of an amide bond with Lys residues on VLPs. This simple method has been used to conjugate Zika virus–derived E‐DIII protein to the immunologically improved CuMV_TT_‐VLPs, which induced neutralizing antibodies without enhancing Dengue virus infection.^59^ Furthermore, canine IL‐31 was conjugated to CuMV_TT_‐VLPs to treat chronic itch in dogs. Several VLP‐based vaccines in preclinical and clinical studies are based on these simple chemical methods.^20^, ^60^ However, for the development of a personalized VLP‐based vaccine in cancer settings, we have recently used Cu‐free click chemistry method to couple peptides to CuMV_TT_‐VLPs or Qβ‐VLPs. The method showed efficacy and induced specific T cell response protective against tumor progression in an aggressive transplanted melanoma murine model.^61^, ^62^ This chemistry may be favorable over SMPH as Cys within T cell epitopes may react with SMPH resulting in epitope inactivation. Additionally, non‐reacting SMPH may be toxic, requiring complex purification processes which may not be optimal for rapid generation of personalized cancer vaccines. Cu‐free click chemistry is a bio‐orthogonal chemistry^63^ and is commercially available, in the form of, for example, dibenzocyclooctyne‐NHS ester. This cross‐linker reacts with Lys residues on VLPs to incorporate a cyclooctyne moiety which reacts with an azide‐labeled molecule to form a stable triazole bond without a Cu catalyst. This method enables rapid, safe, and efficient coupling for good manufacturing practice–produced modified VLPs.

**TABLE 2:** CuMV_TT_‐VLPs as a vaccine platform

Ligand	Target species	Disease/condition	Reference
IL‐5	Horse	Insect‐bite hypersensitivity	64, 65
IL‐31	Dog	Atopic dermatitis	66
Fel d 1	Cat	Allergic symptoms in humans, facilitating owner‐cat interactions	67
NGF	Mouse	Pain in osteoarthritis	68
Ara h 2	Mouse	Peanut allergy	69
E‐DIII	Mouse	Zika virus infection	59
IL17A	Mouse	Psoriasis	55
Gp33 as a model antigen	Mouse	Melanoma	62
TRAP	Mouse	Malaria	70

Abbreviation: CuMV, cucumber mosaic virus; NGF, nerve‐growth factor; TRAP, thrombospondin‐related adhesive protein; VLPs, virus‐like particles

Many antibacterial vaccines are based on conjugation of carbohydrates to carrier proteins. VLPs are obvious candidates for such carrier proteins as they enhance the immunogenicity of conjugated antigens greatly.^71^ However, production of bacteria‐derived carbohydrates is expensive and comes with a number of obstacles, for example, growing large amounts (kg) of dangerous bacteria. We therefore consider it a major breakthrough that it has recently been possible to glycosylate VLPs in vivo with defined bacterial carbohydrate structures in the cytoplasm of *E coli*. Yields of such VLPs are high, and the cost of goods is in orders of magnitude lower than conventional production.^71^ Due to the low cost of goods, this opens up the possibility to immunize farm animals against bacterial infections using VLPs displaying carbohydrates. This may represent a major step for the one‐health concept and may allow to substantially reduce antibiotic resistance spread by farm animals treated with antibiotics.

### 2.3 VLPs and nucleic acids (NAs)

**FIGURE 3:**
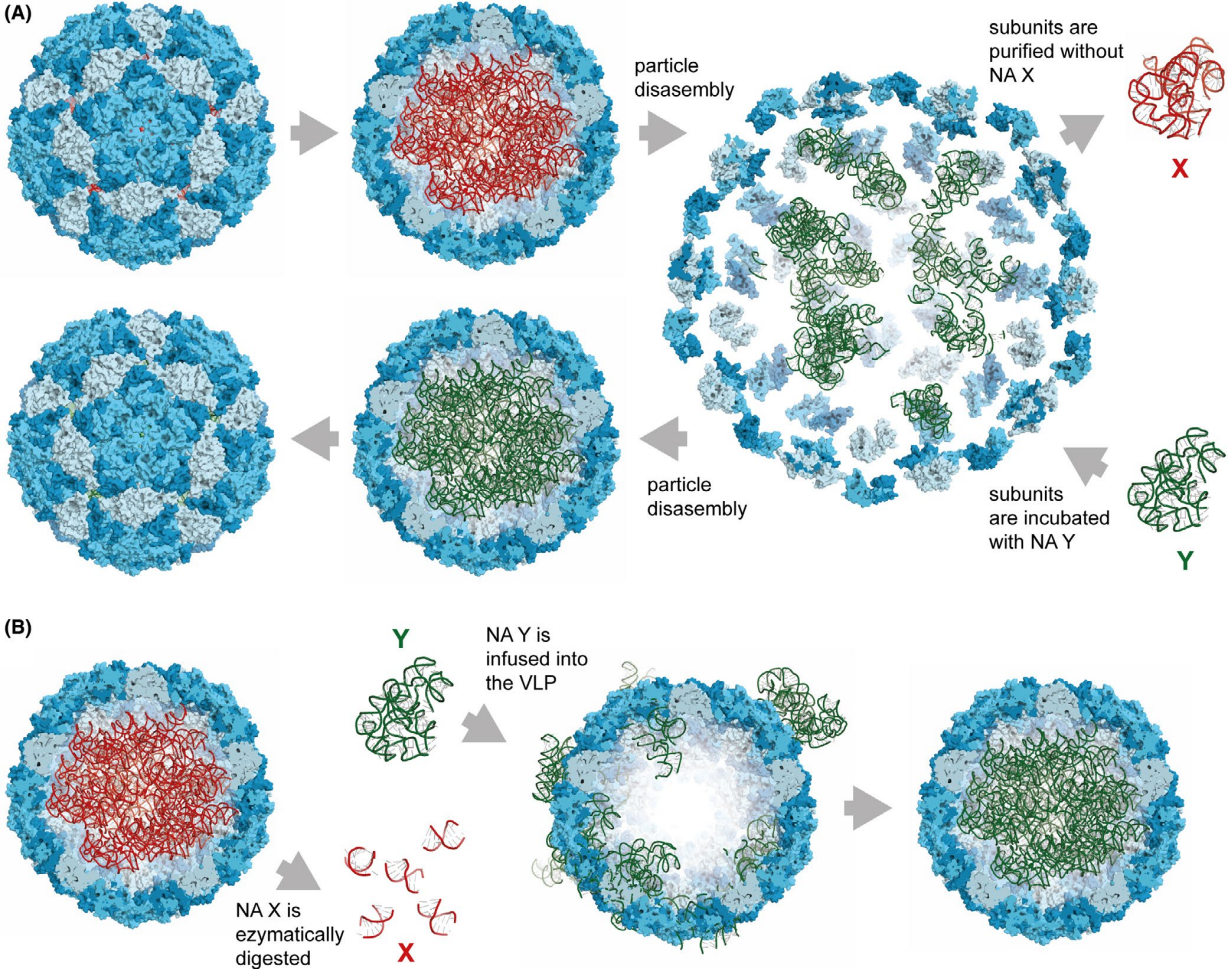
Virus‐like particle (VLP) NA exchange. Two methods for exchanging VLP nucleic acids (NAs). A, Particle disassembly and reassembly. In this method, the particle is initially disassembled, the endogenous NA is removed, and a new NA is repackaged into the VLP during the reassembling process. In the order indicated by the arrows, the images represent the exterior of the particle at the beginning of the process; the interior of the particle packaged with endogenous NA “X”; disassembled particle being reassembled, after removal of NA “X,” in the presence of exogenous NA “Y”; the interior of the particle packaged with NA “Y”; and the external view of the particle at the end of the process. B, Enzymatic digestion and infusion. Instead of being washed out after the particle disassembling, in this method the endogenous NA is eliminated by enzymatic digestion. The exogenous NA is then repackaged into the VLP by diffusion, which is facilitated by the high porosity of the particle's surface. In the order indicated by the arrows: the interior of the particle packaged with endogenous NA “X”; digestion of NA “X” and diffusion of exogenous NA “Y” into the VLP; and the interior of the particle packaged with NA “Y.” Images generated from unrelated protein data bank (PDB) files 5KIP and 1Y0Q

VLPs can assemble on a polyvalent scaffold of NA, usually RNA. Generally, the CP of viruses play a role in organizing the packaged NA; conversely, NA plays a role in structure's assembly and immunogenicity of VLPs. A highly ordered NA structure can be seen in bean pod mottle virus,^72^ tobacco necrosis virus,^73^ or nodaviruses.^74^ Bacterial expression systems such as *E*. coli is the most widely used system for the production of non‐enveloped VLPs. VLPs self‐assemble on prokaryotic RNA (pRNA) during the expression process.^75^ By way of example, CuMV_TT_‐VLPs can successfully be expressed in *E* coli host. Cryo‐electron microscopy analysis of the VLPs demonstrates additional electron density in its interior cavity. This ordered structure most likely reflects the tightly bound host pRNA suggesting its role in stabilizing the subunits of the VLPs.^55^ Assembly studies are vital to understanding virus formation in biological settings which facilitates the packaging of non‐viral materials for VLP‐based vaccine development. Flock house virus (FHV) was used to study the encapsidation preference of specific RNA via next‐generation sequencing experiments. It has been shown that the native viral particle contains 1% host‐derived RNA and 99% viral genomic RNA. In contrast, recombinant FHV‐VLPs can encapsidate a larger percentage of host RNA in insect cell culture.^38^ Other VLPs have been shown to encapsidate different host RNAs, including messenger (mRNA), transfer (tRNA), or ribosomal (rRNA), as well as RNA encoding the CP.^76^ The addition of a potent vaccine adjuvant is an essential strategy to activate antigen‐presenting cells (APCs) mainly dendritic cells (DCs) for induction of T cell responses. Therefore, the interior surface of VLPs is often exploited to package different TLR ligands such as ssRNA, dsRNA, or CpGs.^77^, ^78^ To achieve this, the host NA has to be removed first. This is typically accomplished by the disassembly/reassembly method (Figure 3A) or by the enzymatic digestion method of the host NA and repackaging with the desired one (Figure 3B).

A recent study performed by us has used the disassembly/reassembly method to compare three different types of RNA packaged inside Qβ‐VLPs displaying the M2 ectodomain from influenza virus as an antigen. pRNA, eukaryotic RNA (eRNA), or tRNA has been used, and the results indicated that the type of packaged RNA can alter the induced protective humoral immune response in a murine influenza model. Specifically, pRNA packaged in Qβ‐VLPs induced the most protective IgG2b/c, while eRNA showed more shift to IgG1 with less protection. The study provided evidence that the shifting of the Ab response is dependent on TLR7 and it rules out the role of the cytoplasmic mitochondrial antiviral signaling protein (MAVS) and retinoic acid–inducible gene‐like receptor (RIG‐1). Such findings are crucial for rational design of VLP‐based vaccine aiming at inducing the most desirable and strong B cell response.^79^

The second method is the enzymatic digestion method, which is achieved by treating the VLPs with RNase to remove the RNA. Subsequently, the empty coat VLP can be repackaged with small oligonucleotides of choice, yielding a loaded VLP. This method has been used in different studies utilizing the bacteriophage Qβ or HBcAg.^61^, ^80^, ^81^ Alternatively, the desired NA can be packaged in VLPs during the cultivation process of the recombinant protein in the host system. Polyomavirus JC VLPs were tested to package a model plasmid of 9 kb directly when expressed in *E coli* cells.^82^ VLPs, however, are usually poor vehicles for transgene expression, as they are degraded in endosomes and DNA may not easily reach the cell nucleus. Indeed, CpGs and RNA are ideal cargo, as they are sensed by TLR9 and TLR7/8 localized endosomes, where the ligands are released after protein shell degradation.

## 3 DELIVERY

### 3.1 Routes of administration

The protection conferred by a VLP‐based vaccine is essentially dependent on the induced humoral and/or cellular immune response. However, the science of vaccine administration route is a poorly developed and described area. This is mainly due to the fact that vaccine trials lack standardized comparison of the injection site, needle length, or injection techniques.

Evidence‐based medicine aims at improving the quality of health methodologies. Thorough careful assessment of published clinical data regarding the route of vaccine administration subcutaneous (SC) vs intramuscular (IM) revealed that the current practice is based on tradition rather than clinical data.^83^, ^84^ Traditionally, vaccines were injected SC until it was noticed that adjuvanted vaccines such as Alum could induce unacceptable local reactions at the injection site in humans and injections were changed to IM, most probably because the injection site reactions were less visible. Indeed, the four licensed VLP‐based vaccines as well as the first licensed malaria VLP‐based vaccine RTS,S (Mosquirix^™^) use IM route of administration as shown in Table 3.

**TABLE 3:** Route of immunization of the licensed VLP‐based vaccines.

VLP vaccine	License date	Route of immunization
Gardasil	USA, 2006	IM
Cervarix	USA, 2009	IM
Recombivax	USA, 1983	IM
Hecolin	China, 2011	IM
Mosquirix^™^	EMA, 2015	IM

Abbreviations: IM, intramuscular; SC, subcutaneous; VLP, virus‐like particle

When comparing SC to IM routes of vaccine injection, IM has shown a faster rate of absorption of administered materials.^85^ This is due to the fact that muscles are more abundant with larger blood vessels than SC tissues which have lower vascularity. This may result in slower mobilization of the vaccine and thus slower processing of the antigen.

Under normal non‐inflamed conditions, very few resident immune cells such as APCs are found in muscles.^86^ Administering vaccines such as VLPs induces a transient pathological condition in the muscle resulting in infiltration by immune cells such as macrophages, neutrophils, DCs, and lymphocytes.^87^ Additionally, myocytes also react to the pathological condition by expressing different cytokines and chemokine receptors such as IL‐6, IL‐1, IFN‐γ, CCR2, CCR4, and CCR10.^88^ Such transient reactions rarely cause serious adverse effects at the injection site. In a series of 26,294 adults, of whom 46% received an IM injection, only 0.4% had a local injection site reaction.^89^ On the other hand, SC injections can result in abscesses and granulomas in humans.^90^, ^91^, ^92^, ^93^ However, in preclinical studies with rodents, the SC is more commonly used route of injection than IM. This is because of the simplicity of the method and the possibility of delivering larger volume of the vaccine and more freedom in choosing the injection site.^94^ Additionally, using the SC route for VLP‐based vaccines did not result in any adverse effects in murine models. Regarding the induced immune response of a VLP‐based vaccine when comparing the SC vs IM route of injection, we have previously shown that the SC route of injection is effective at inducing a protective B or T cell response in a wide range of preclinical studies.^61^, ^64^, ^68^, ^95^, ^96^, ^97^ Furthermore, the SC and IM routes of administration of an experimental VLP‐based vaccine in humans resulted in similar antibody (Ab) titers.^98^ These data indicate that SC route of immunization may also have its merits.

There is evidence that intradermal vaccination may be superior to other routes of immunization. Indeed, a study published in 2009 has used a near‐infrared fluorescent dye (NIR) coupled to simian‐human immunodeficiency virus (SHIV)–like particles to track their movement in SKH‐1 mice using different immunization routes. The study has observed that intradermal immunization with SHIV‐VLPs involved a broader range of lymph nodes (LNs). This resulted in the production of the highest Ab titers confirmed by the increase in germinal centers in the spleen as well as the highest antigen‐specific cytotoxic T‐lymphocyte (CTL) response.^99^ Similar findings were observed in humans using influenza virus vaccines, where intradermal immunization retained full immunogenicity even at up to five times lower vaccine dose.^100^ In contrast to Ab responses, we have compared the induction of MelanA‐specific T cells in a phase I/II study for stage II‐IV melanoma patients upon SC vs intradermal vs intranodal injection and found no difference in responses.^60^

With the advent of oncolytic viruses injected directly into the tumor, this route is also investigated for VLPs. The bacteriophage Qβ‐VLPs filled with A‐type CpGs (originally called by us QbG10 now CMP‐001) have been used for intratumoral injections and are currently being examined in combination with systemic anti‐PD‐1 checkpoint inhibition in two different clinical trials for patients with advanced melanoma who showed resistance to anti‐PD‐1 treatment. The recently collected data from the ongoing trials show manageable toxicity and potential to reverse the resistance to anti‐PD‐1 (NCT02680184/NCT03084640). Lizotte et al have investigated a further route of application and shown that inhalation of cowpea mosaic virus (CPMV)–like particles caused regression of an established B16F10 lung melanoma by generating an efficient systemic anti‐tumor immune response. The study has shown that an important player of the generated anti‐tumor response in the tumor microenvironment was neutrophils which could rapidly take up CPMV‐VLPs.^101^ Furthermore, the authors have shown that intratumoral injection of CPMV‐VLPs is also effective in treating dermal B16F10 melanomas, forming central tumor necrosis.^101^

A combination of different routes has also been investigated using human immunodeficiency virus (HIV)–like particles which resemble the virus envelope protein in its native conformation. It has been shown that that priming with HIV‐VLPs intranasally followed by sub‐cheek route for boosting resulted in the highest titers of specific immunoglubulin B (IgG) compared to the other combination of priming and boosting routes used (intradermal‐intranasal‐sub‐cheek).^102^

Mucosal Ab responses may be an interesting case where the route of immunization is concerned. SC immunization of mice with RNA‐loaded VLPs leads to strong systemic immunoglobulin M (IgM), immunoglobulin A (IgA), and IgG responses. While the IgG response is T_H_ cell–dependent, both IgM and IgA antibodies are T_H_ cell–independent.^103^ In contrast, mucosal IgA responses in the lung were absent while strong mucosal IgG responses are observed. Mucosal immunization, on the other hand, results in strong mucosal IgA and IgG responses. IgA responses are heavily dependent or TLR7/8 or TLR9 ligands packaged in VLPs for mucosal and systemic IgA. Again, systemic and mucosal IgA responses are regulated differently, as systemic IgA requires TLR7/8 or TLR9 in B cells, while mucosal IgA responses do not require TLR7/8 or TLR9 in B cells but DCs rather, which induce IgA in a Transforming growth factor beta (TGF‐β)‐ and BLyS‐dependent fashion.^104^ Hence, systemic IgA responses are similarly regulated as subclass switching to IgG2a as they also require TLR expression in B cells rather than DCs.^105^ Recently, it has been shown that administering the influenza VLP vaccine (M2e5x VLP) intranasally facilitated mucosal delivery of the vaccine and can induce a broad cross‐protection, prevent weight loss, lower the viral load, attenuate the inflammatory reaction and induce germinal center formation. The study indicated that the observed protective role is managed by B cells as well as CD8^+^ and CD4^+^ T cells.^106^

In conclusion, it seems that VLPs efficiently reach lymphoid organs from most injection sites and prime similar but not always identical immune responses.

## 4 DYNAMICS

As discussed above for the delivery, rules for induction of Ab vs T cell responses may differ and both design and delivery of VLPs have important consequences for the dynamics of the immune response.

### 4.1 VLP‐based vaccine formulation

VLP‐based vaccines are made of a restricted number of antigens or individual components of the targeted pathogen, hopefully able to confer a protective and/or a therapeutic effect. Accordingly, the protective epitope must be displayed in its native form, at least if the induction of Abs is the desired response. This may be different for T cell‐based vaccines, where native display of the epitope is not required. Furthermore, VLP‐based vaccines must be optimized for antigen density, dose, and prime/boost interval to obtain a potent Ab response. Unexpectedly, a recent study has demonstrated that such criteria are not necessary for achieving high‐avidity T cell responses.^107^ Indeed, requirements for vaccines designed to induce Abs (most prophylactic vaccines and vaccines targeting endogenous molecules for their blockage) differ from vaccines aiming to induce T cell responses. In contrast to vaccines against simple viruses, most vaccines targeting complex pathogens will need to induce B and effector T cells. Table 4 summarizes design requirements for optimal delivery to cause a dynamic response of desired specificity.

**TABLE 4:** Requirements for optimal induction of Ab or T cell responses or both

	Antibody (Ab)	T cells	Antibody (Ab) and T cells
Antigen	Native display	Irrelevant	Native display
VLP scaffold important for	Repetitive display	Particulate for DC targeting	Both
Adjuvants	Activation of B cells (and DCs)	Activation of DCs	Both
Adjuvants formulation	Physically linked to VLPs	Formulated in depot and/or codelivered with adjuvant^33^, ^127^	Physically linked to VLPs and formulated in depot adjuvants
Depot formation	Not very important as antigen persists on FDCs	Depot for long‐term stimulation	Depot for long‐term stimulation; importantly, the adjuvants may not compromise antigen structure
Size	<200nm for effective drainage to LN	Antigens may be larger	<200 nm for effective drainage to LN

Abbreviation: DCs, dendritic cells; Follicular dendritic cells (FDCs).

### 4.2 B cells and antibodies

B cell responses are optimally stimulated by repetitive particles displaying native epitopes and which are associated with a potent adjuvant for B cells. VLPs loaded with RNA or CpGs and displaying full‐length antigens on their surface are therefore ideal for induction of Ab responses. Depot formation may be less important for vaccines aiming to induce Abs, since VLPs are recognized by natural IgM antibodies which recruit C1q and lead to deposition on follicular DCs (FDCs).^108^ FDC‐associated antigen is essentially an endogenous antigen depot that can persist for weeks and months.^109^ As discussed above, RNA or CpG‐loaded VLPs trigger strong IgG2a and IgA responses but require TLR expression in B cells, not DCs.^104^, ^110^ Hence, adjuvants for B cells should be physically associated with the VLPs to recognize antigen and trigger B cells directly. As discussed earlier, the type of RNA packaged in VLPs is important to enhance B cell responses. We found that pRNA was clearly superior to eRNA or tRNA. Cytosolic sensor RIG‐I and MAVS had no influence on the magnitude of the response, and TLR7 itself may be able to distinguish between prokaryotic and eukaryotic RNA.

Conventionally, vaccines aim to induce long‐lived Ab responses rather than memory B cells.^111^ We have recently shown that memory B cells that have been induced in the presence of TLR7 stimulation can rapidly differentiate into secondary plasma cells which produce very high levels of Abs.^112^, ^113^ Again, TLR7‐stimulation was necessary to cause differentiation of memory B cells into secondary plasma cells. The response of secondary plasma cells peaked within a few days after antigenic challenge and produced a very rapid first wave of Abs. Interestingly, these secondary plasma cells were not long‐lived and did not differentiate into a new wave of memory B cells. In contrast, they essentially all died within 6 days. Indeed, all memory B cells generated during the secondary response were derived from naive B cells. Hence, a first wave of antibodies is produced very rapidly but the immune system mounts a primary B cell response under the protective shield of the secondary plasma cell–derived Abs.^114^ This ensures that original antigenic sin is kept minimal and allows the immune system to optimally respond to slightly evolved, re‐emerging pathogens that may not be optimally recognized by the existing Ab specificities. Hence, induction of memory B cells with the ability to rapidly differentiate to secondary plasma cells may be an important, currently underestimated goal of vaccination.

### 4.3 T cell responses

A VLP‐based vaccine displaying T cell epitopes can elicit a strong T_H_ 1 as well as CTL responses despite the fact that they do not carry any genetic material for replication.^115^, ^116^, ^117^, ^118^, ^119^, ^120^ The particulate nature of VLPs allows them to be cross‐presented on major histocompatibility class I (MHC‐I) molecules as well as on major histocompatibility class II (MHC‐II) for effective priming of CD8^+^ and CD4^+^ T cells, *respectively*.^121^, ^122^ As mentioned earlier, the type of NAs packaged in the VLPs plays a vital role in determining the desired immune response. Non‐methylated CpGs, TLR9 ligands, are potent stimulators of the innate immune system characterized by upregulating costimulatory molecules, cytokines, and chemokines.^123^ Our recent study has investigated the transcriptional signature in DCs from mice vaccinated with Qβ packaging TLR7/8 or TLR9 ligands and displaying H‐2D^b^ gp33 epitope. The most striking observation involved CCL2 chemokine which was distinctly expressed in DCs 24 hours following immunization with Qβ(type‐B CpGs)‐p33, a potent TLR9 ligand. The identified pathway is thought to play an important role in DCs activation and subsequent induction of potent (CTL) response.^124^

Administering synthetic CpGs directly in vivo may result in unfavorable outcomes such as toxic shock, auto‐Ab production, inflammation, or the induction of anti‐DNA antibodies.^125^ Such obstacles can be easily overcome by packaging CpGs in VLPs which indeed reduces side effects and results in an efficient CTL response.^20^, ^81^, ^119^ The dogma for an optimal response in VLP‐based vaccines is that both antigen and adjuvant should be delivered in the same VLP.^81^, ^126^ However, we have shown that this is not always necessary; adjuvants such as CpGs can be packaged in separate VLPs and mixed with the vaccine prior to administration without the need of physical linkage and would still generate a strong CTL response.^33^ Similar results were obtained when admixing E7 protein oligomers derived HPV with Qβ‐VLPs loaded with CpGs.^127^ The formula could induce a protective CD4^+^ and CD8^+^ T cell response in a HPV mouse model. These findings indicate that physical linkage of both antigen and adjuvant in a VLP‐based vaccine may not be necessary for effective T cell activation. In contrast, simple, admixing VLPs did not enhance B cell responses, indicating the rules for T and B cell responses are different (see also Table 4).

Adjuvants may enhance the immune response by several mechanisms such as prolonging the release of the antigen at the injection site and upregulating different cytokines, chemokines, and costimulatory molecules. This results in increased maturation and antigen uptake by APCs for effective presentation on MHC‐II or by the activation of inflammasomes and TLRs.^128^ TLR agonists packaged inside VLPs are recognized by pattern recognition receptors (PRRs) while particulate adjuvants such as Alum are considered damage‐associated molecular patterns (DAMPs). As discussed above, adjuvants may also act directly on B cells, as, for example, RNA and CpGs stimulating TLR7/8 and TLR9, respectively.

We have recently harnessed the physiological properties of the lymphatic system by formulating CuMV_TT_‐VLPs displaying H‐2D^b^ gp33 peptide derived from lymphocytic choriomeningitis virus with a biodegradable microcrystalline tyrosine depot adjuvant (MCT). CuMV_TT_‐VLPs are nanoparticles with packaged RNA as TLR7/8 agonist while MCT is a micron‐sized depot‐forming adjuvant capable of activating the inflammasome. Such formulation has increased the resultant specific CTL response in a murine melanoma model.^62^ The novel immune enhancer can also be translated to humans as VLPs used are well defined and the micron‐sized adjuvants have been used for decades in allergen‐specific desensitization.^129^ This micron‐/nano‐hybrid system harnesses the properties of the lymphatic system optimally, as the micron‐sized MCT cannot enter the lymphatics and only the slowly released nanoparticles are actually able to do so, resulting in a slow‐release depot of VLPs.

## 5 CONCLUDING REMARKS

The 3Ds of vaccinology, design, delivery, and dynamics, are 3 key components for the efficient generation of a protective vaccine. Optimal design allows repetitive display of native antigens on VLPs loaded with TLR ligands for direct stimulation of B cells for IgG and IgA production as well as enhanced T cell responses. Optimal delivery may be key for inducing mucosal responses, or enabling dose sparing and mediating direct oncolytic activity if applied into the tumor. Optimally designed and delivered vaccines will result in optimally dynamic immune responses, with the desired outcome of strong T cell responses, highly specific Ab responses, and optimal clinical efficacy.

## CONFLICT OF INTEREST

The authors declare no conflict of interest.

## Supporting information

Video S1
